# Frontiers and hotspots in hand, foot, and mouth disease research during 2006 to 2023: A bibliometric and visual analysis

**DOI:** 10.1097/MD.0000000000038550

**Published:** 2024-06-14

**Authors:** Yunzhi Li, Ying Ruan, Xiangjie Zhai, Junjie Ye, Yujie Xiao, Jiawei Liang, Ni Zhu

**Affiliations:** a Key Laboratory of Environmental Related Diseases and One Health, School of Basic Medicine Sciences/School of Pharmacy/National Demonstration Center for Experimental (General Practice) Education, Xianning Medical College, Hubei University of Science and Technology, Xianning, China.

**Keywords:** bibliometrics, enterovirus, hand, foot, mouth disease, vaccine

## Abstract

**Background:**

Enteroviruses-infected hand, foot, and mouth disease (HFMD) seriously threatens human health. This study aimed to analyze the research status, hotspots, and frontiers of HFMD.

**Methods:**

Publications on HFMD between January 1, 2006, and January 31, 2023, were retrieved from the Web of Science Core database. Bibliometric tools, including CiteSpace, VOSviewer, R package “Bibiometrix,” SCImago Graphica, and Charticulator, were utilized to analyze and visualize the data.

**Results:**

A total of 1860 articles from 424 journals, involving 8815 authors from 64 countries and 1797 institutions were analyzed. The number of studies on HFMD has shown an increasing trend over the past 18 years, with an annual increase observed since 2006, which is particularly prominent after 2010. Research in this field has centered on the Asian region. Notably, the research hotspots were mainly focused on vaccines, epidemiology, and pathogenesis of HFMD. Among the researchers in this field, Zhang Yong emerged as the most prolific author, while Xu Wenbo had the most significant influence. The Chinese Academy of Sciences was the most productive institution, and China was the most productive country for HFMD research.

**Conclusion:**

By bibliometric analysis, researchers in the HMFD field can efficiently identify and visually represent their research focus and limitations. In the future, it is crucial to maintain ongoing surveillance of HFMD outbreaks and their pathogenic changes. Additionally, future research should extensively explore the molecular mechanisms underlying Enteroviruses-induced HFMD with a focus on developing vaccines and therapies.

## 1. Introduction

Hand, foot, and mouth disease (HFMD) is an infectious disease caused by enteroviruses that primarily affects children under 5 years of age. Since 2008, HFMD has been classified as a Class C infectious disease in China.^[1]^ Although most cases of HFMD are mild and self-limiting, some children may develop severe complications. Usually, HFMD has an incubation period of 2 to 10 days, and its symptoms include fever and rash on the hands, feet, mouth, and buttocks.^[2]^ The progress of HFMD can be divided into 5 stages. Most patients go through the first stage and recover within 1 week. However, some patients may have severe symptoms, such as high fever, nervous system involvement, and abnormal respiratory or circulatory function. Atypical manifestations may also occur, and differential diagnosis is necessary to distinguish HFMD from other diseases. Moreover, recent studies have found that patients who have recovered from severe HFMD may experience severe long-term neurological complications.^[2]^ Therefore, HFMD has emerged as a major public health concern.

Human enteroviruses (EVs) cause HFMD and belong to the Enterovirus genus of the Picornaviridae family. Since 1999, these viruses have been classified into 4 categories based on their molecular, biological, and genetic characteristics. More than 100 types of EVs have been identified around the world. Enterovirus 71 (EV71) and Coxsackievirus A16 are the most common causes of HFMD. Currently, some other EVs, such as CVA6 and Coxsackievirus A10 (CVA10), have also played an important role in HFMD outbreaks. The recent identification of Tomato flu, an illness similar to HFMD caused by an Enterovirus in India, has rekindled people’s interest in HFMD outbreaks.^[3]^ Although these Enteroviruses can trigger a strong immune response, there is little cross-immunity between different serotypes.^[2]^ Therefore, there is an urgent need to develop a multivalent vaccine to enhance protection against various EV serotypes and reduce the risk of HFMD outbreaks.^[4]^ Thus, there are still many unresolved issues regarding HFMD.

Bibliometrics employs mathematical and statistical techniques to collect data on publishing rates, characteristics, and patterns in a specific field. Through analyses of authors, co-occurrence, and citations, we can show research trends and status in specific fields. This type of research can map the field of knowledge and determine the productivity of researchers, internal collaboration, and distribution, as well as that of affiliated institutions and journals. Additionally, bibliometric research revealed research hotspots in a field and possible future development directions. This study aimed to summarize the research status and development stage of HFMD and its main pathogenic viruses, including epidemiology, molecular biology, clinical treatment and diagnosis, and the development and application of vaccines to provide suggestions for clinical treatment and scientific research.

## 2. Methods

### 2.1. Data collection and search strategy

The data for this study were obtained from the Web of Science Core Collection (WoSCC), which includes 6 indexes: Science Citation Index EXPANDED (SCI-EXPANDED), Social Sciences Citation Index (SSCI), Arts & Humanities Citation Index (AHCI), Emerging Sources Citation Index (ESCI), Current Chemical Reactions (CCR-EXPANDED), and Index Chemicus (IC). The search was conducted from January 1, 2006, to January 31, 2023, using the search query Topic Sentences (TS) = (“Hand, foot, and mouth disease “OR” HFMD “) AND TS= (“ Enterovirus “OR” coxsackievirus “OR” echovirus “). Only journal articles and reviews written in English were retained. A total of 1860 articles were included in the final analysis.

### 2.2. Bibliometric analysis

The bibliometric tools, including CiteSpace, VOSviewer, R package “Bibiometrix,” and SCImago Graphica, were used for data analysis and visualization. According to previous methods, we exported all records from the WoSCC database in plain text format and saved them as “download_xxx.txt” files.^[5,6]^ Subsequently, these files were imported into CiteSpace 6.2.R4 software to identify critical scientific information, including keyword bursts, reference bursts, and journal dual-map overlays.^[7,8]^ The parameters of the CiteSpace software included the time span from January 1, 2006, to January 31, 2023, with 1 year per slice. Node types included keywords and references. The selection criteria were set to the top 50 for each slice, while the other settings remained the default. The parameters in VOSviewer were set as follows: select “association strength” as the standardization method, and set the thresholds as at least 5 publications in countries/regions, at least 30 publications in institutions, at least 20 publications by author, publications, or co-citations by journal, at least 50 co-occurring keywords, and at least 100 citations in each publication.^[9,10]^ The data exported from the VOSviewer analysis were imported into Charticulator (https://donghaoren.org/charticulator/index.html) and SCImago Graphica to optimize the visual outputs while maintaining their original contextual meanings. Charticulator was used to enhance author co-occurrence networks and journal co-occurrence networks, while SCImago Graphica perfected countries’ analysis and keyword co-occurrence analysis.^[11,12]^ Furthermore, we used the R 4.4.2 package Bibiometrix to map the annual scientific output and analyze the geographical distribution of publications over a period of time.^[13]^ Finally, Microsoft Excel and Word were used for data sorting and statistics, and tables and H-index of the top 10 countries were made, as well as the chart of each article cited and the total cited.

## 3. Results

### 3.1. Basic information

**Figure 1.**
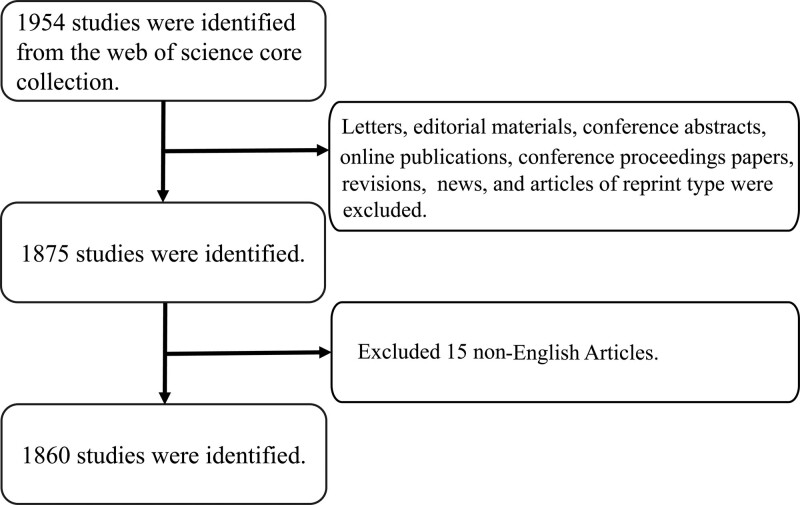
Flowchart of screening strategy.

According to the screening strategy shown in Figure 1, we obtained 1860 HFMD-related publications for further analysis. Of these 1860 articles, 93% were articles, while the rest were listed in the reviews (n = 134, about 7%). All the included articles were cited 44,206 times, each article was cited 23.77 times, and the H-index was 87. The articles were published in 64 countries/regions, 1797 institutions, 8815 authors, and 424 journals.

### 3.2. Annual publication trends

**Figure 2.**
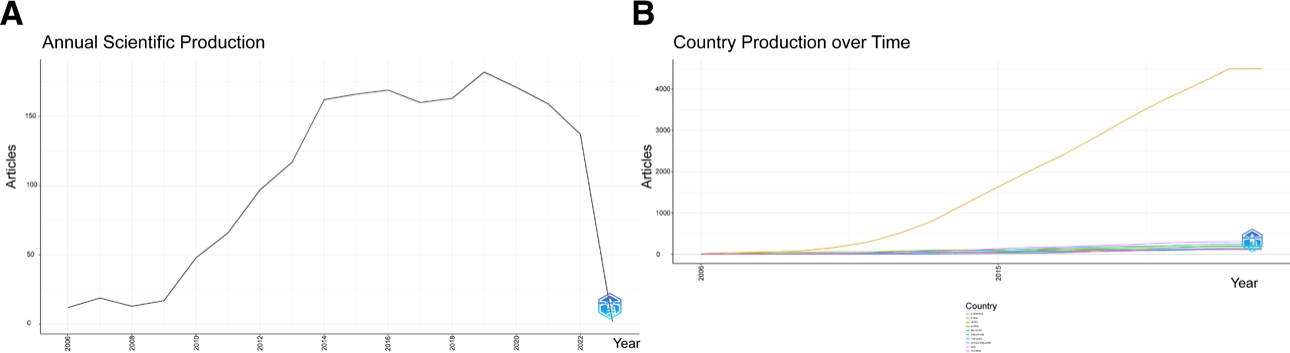
The annual scientific yield of HFMDs. (A) Annual HFMD publication; (B) top 10 HFMD-producing countries yield over time.

In order to analyze the annual publication trends from 2008 to 2016, as shown in Figure 2A, the global research on HFMD shows a consistent growth from 2008 to 2016. Following this period, the publication output was maintained at a stable level until 2018, reaching a peak of 190 articles published in 2019. This remarkable increase can be attributed to the rising global epidemic of HFMD, especially in China, Vietnam, and Thailand, where the incidence and mortality of HFMD are staggering. As a result, an increasing number of researchers are forced to undertake relevant research and publish their findings. Nevertheless, since 2021 and 2022, the number of publications has exhibited a declining trend, but it is expected to reach a stable level or may increase in the future. Figure 2B demonstrates the annual changes in the number of HFMD-related publications for the top 10 countries with the highest publication output. Since 2006, the number of publications on HFMD in China has greatly increased, surpassing those in other countries. Furthermore, this trend is expected to continue to develop steadily after 2022. In contrast, the output of publications in other countries remains stable, though low. As the largest country in Asia and the area most seriously affected by HFMD, China has experienced a series of HFMD outbreaks since the first outbreak of HFMD in 2006. This has prompted domestic researchers to do more research on the characteristics and effects of this disease.

### 3.3. Geographical distribution of publications

**Figure 3.**
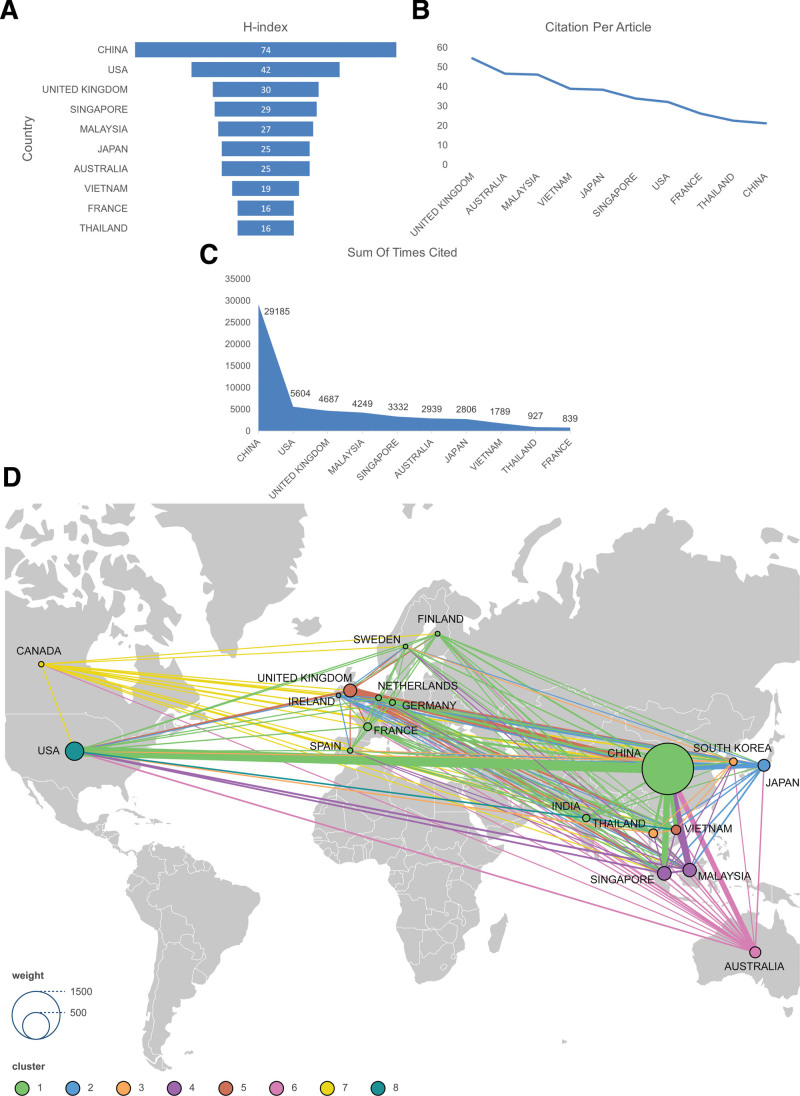
Visual analysis of the country with HFMD publications. (A) Index for the top 10 countries with HFMD articles; (B) average citations of articles from the top 10 HFMD countries; (C) total citations for the top 10 HFMD articles; (D) collaborative graph of at least 10 countries with HFMD articles.

In total, 64 countries conducted HFMD research. We calculated the H-index, average citations, and total citations of the top 10 countries with the largest number of articles published from 2006 to 2023 (Fig. 3A). China, the United States of America (USA), and the United Kingdom (UK) were the top 3 countries with the highest H-index, while each article in the UK, Australia, and Malaysia had the highest average citations (Fig. 3B). The countries with the highest total citations were China, the USA, and the UK, as shown in Figure 3C. Figure 3D shows the map of collaborations between countries with at least 10 publications. Geographically, cooperation in virology research is spread worldwide, and research locations are concentrated in Asia, while China is in the leading position in virology research. China has collaborated with several countries, with collaboration with the USA being the most substantial. Countries of different colors on the map have established cooperative relations, while countries of the same color have stronger and more remarkable cooperative relations with each other. In particular, China has established cooperative relations with the USA, Malaysia, the UK, Australia, and Singapore.

### 3.4. Visual analysis of institutions and authors

**Figure 4.**
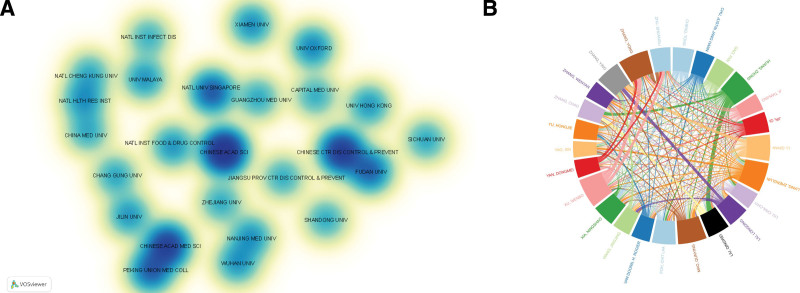
Analysis of institutions and authors of HFMD-related publications. (A) Number of institutions with >30 HFMD publications; (B) authors with >20 HFMD publications.

The visualization of the institutions is presented in Figure 4A, which shows the cooperation heat map between the institutions. We presented a total of institutions with more than 30 articles. The heat map illustrates that Fudan University closely cooperated with the Chinese Center for Disease Control and Prevention, and the same close link was also observed between the Chinese Academy of Medical Sciences and Peking Union Medical College. The deepest color indicates these institutions made great contributions. Table 1 shows that among the top 10 institutions that published the most publications, the Chinese Academy of Sciences ranked first with 148 articles, followed by the Chinese Center for Disease Control and Prevention (115), and the Chinese Academy of Medical Sciences (106). Among the top 10 institutions, the Chinese Center for Disease Control and Prevention had the highest total number of citations, while the University of Oxford had the highest average number of citations. The H-index is frequently employed as an effective index to assess academic influence, whereas Total Linkage Strength (TLS) indicates the strength of collaborative relationships.^[14,15]^ The institution with the highest H-index and TLS was the Chinese Center for Disease Control and Prevention. This demonstrates the potential influence of the Chinese Center for Disease Control and Prevention. 8 of the 10 institutions are from China, indicating that Chinese institutions have deeper research in the HFMD field, and have strong field insights.

**Table 1:** The top 10 institutions ranked by number of publications related to HFMD.

Rank	Affiliation	Articles	Total citations	Average citation	Country	H-index	TLS
1	Chinese Academy of Sciences	148	3574	24.15	CHINA	36	10,331
2	Chinese Center for Disease Control and Prevention	115	4479	38.95	CHINA	40	13,482
3	Chinese Academy of Medical Sciences	106	3048	28.75	CHINA	34	6422
4	National University of Singapore	76	2607	34.30	SINGAPORE	26	6679
5	Peking Union Medical College	71	2380	33.52	CHINA	30	4479
6	Fudan University	69	1348	19.54	CHINA	22	5964
7	University of Oxford	50	2165	43.30	UNITED KINGDOM	22	6916
8	Xiamen University	46	733	15.93	CHINA	18	3485
9	National Health Research Institutes-Taiwan	44	1752	39.82	CHINA	24	4494
10	National Institutes for Food and Drug Control, China	43	1855	43.14	CHINA	24	5650

For authors analysis, Table 2 shows that among 1860 articles, 8815 authors and 18,296 co-cited authors participated in the creation of the paper. Among the top 10 authors, Zhang Yong is the most prolific author, publishing 37 papers and leading research on HFMD. Xu Wenbo has the highest total citations, the highest average citations, and the strongest TLS among the authors, which reflects his high-quality output and influence in this field. Nine of the top 10 prolific authors came from China. Mao Qunying and Liang Zhenglun have the highest H-index among authors. The proportion of Chinese authors reflects the interest and enthusiasm of researchers in China for HFMD research.^[16]^ Figure 4B shows Zhang Yong has established close cooperative relationships with Yang Dongmei, Xu Wenbo, and Shuangli Zhu, among other authors, and the establishment of their mutual cooperative relationships intertwine to form a network.

**Table 2:** The top 10 productive authors involved in HFMD research.

Rank	Author	Counts	Total citations	Average cited	Country	H-index	TLS
1	Zhang, Yong	37	1506	40.70	CHINA	16	8124
2	Liang, Zhenglun	36	1553	43.13	CHINA	23	8158
3	Xu, Wenbo	36	1660	46.11	CHINA	18	10,200
4	Mao, Qunying	35	1427	40.77	CHINA	23	9663
5	Huang, Zhong	33	1020	30.91	CHINA	21	7161
6	Li, Qihan	32	903	28.22	CHINA	17	5557
7	Zhang, Ying	30	849	28.30	CHINA	14	5001
8	Poh, Chit Laa	29	785	27.07	SINGAPORE	18	3012
9	Liu, Qingwei	27	535	19.81	CHINA	20	4953
10	Xia, Ningshao	27	828	30.67	CHINA	17	6150

### 3.5. Visual analysis of journals

When analyzing the top 10 journals that published the most articles, we found that they contributed to 33.4% of the articles in this field (Table 3). Except for PLOS ONE and Scientific Reports, the top 10 journals publishing the most articles related to HFMD were all within the field of virology research. PLOS ONE had significantly more publications than other journals, making the largest contribution to HFMD research. The Journal of Medical Virology had the highest impact factor (IF) among the top 10 journals (IF = 12.7 in 2023), indicating higher standards and more persuasive data in journal articles. The Journal of Virology had the highest H-index, indicating its significant influence on HFMD. These journals were mainly concentrated in the third quarter and played a vital role in disseminating research results. Among the top 10 published journals, 4 were from the UK, 3 were from the USA, and the rest were from Austria, the Netherlands, and Switzerland. This demonstrates that the UK and USA are concerned about the field of HFMD.

**Table 3:** The top 10 journals in number of publications related to HFMD.

Rank	Journal	Counts (%)	IF (2023)	H-index	Quartile In category	Countries
1	PLOS ONE	125 (6.72%)	3.7	268	Q3	UNITED STATES
2	Journal of Virology	71 (3.82%)	5.4	271	Q2	UNITED STATES
3	Scientific Reports	66 (3.55%)	4.6	149	Q2	UNITED KINGDOM
4	Archives of Virology	62 (3.33%)	2.7	102	Q4	AUSTRIA
5	Virology Journal	57 (3.07%)	4.8	70	Q3	UNITED KINGDOM
6	BMC Infectious Diseases	56 (3.01%)	3.7	88	Q3	UNITED KINGDOM
7	Vaccine	55 (2.96%)	5.5	164	Q3	UNITED KINGDOM
8	Journal of Medical Virology	46 (2.47%)	12.7	105	Q3	UNITED STATES
9	Antiviral Research	44 (2.37%)	7.6	108	Q2	NETHERLANDS
10	Viruses Basel	40 (2.15%)	4.7	59	Q3	SWITZERLAND

**Figure 5.**
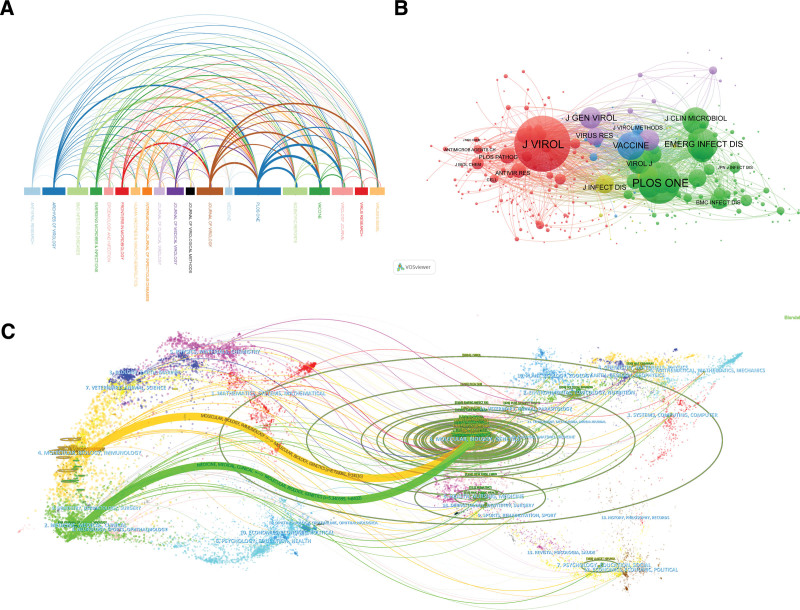
Journal correlation analysis. (A) Journals with > 20 publications related to HFMD; (B) journals with >20 total citations related to HFMD; (C) biplot overlays of HFMD-related journals.

Figure 5A visualizes journals that have published more than 20 articles and cited more than 20 articles, which is consistent with Table 3. The length of the rectangles at the bottom of Figure 5A represents the number of articles published in the indicated journal, and the thickness of the connecting line represents cooperation strength. Figure 5B reveals the core and edge positions of journals with varying article counts. Figure 5C covers a biplot of journals that denotes their topic distribution and research field coverage. The left side of the graph represents the citing journal and the right side of the graph represents the cited journal. The connection paths represent the reference paths, and the linewidth reflects the citation frequency on the z-score scale. The orange and green paths reveal the frequent citations of molecular biology and genetics in molecular biology immunology, medicine, and clinical journals.

### 3.6. Keywords analysis

**Figure 6.**
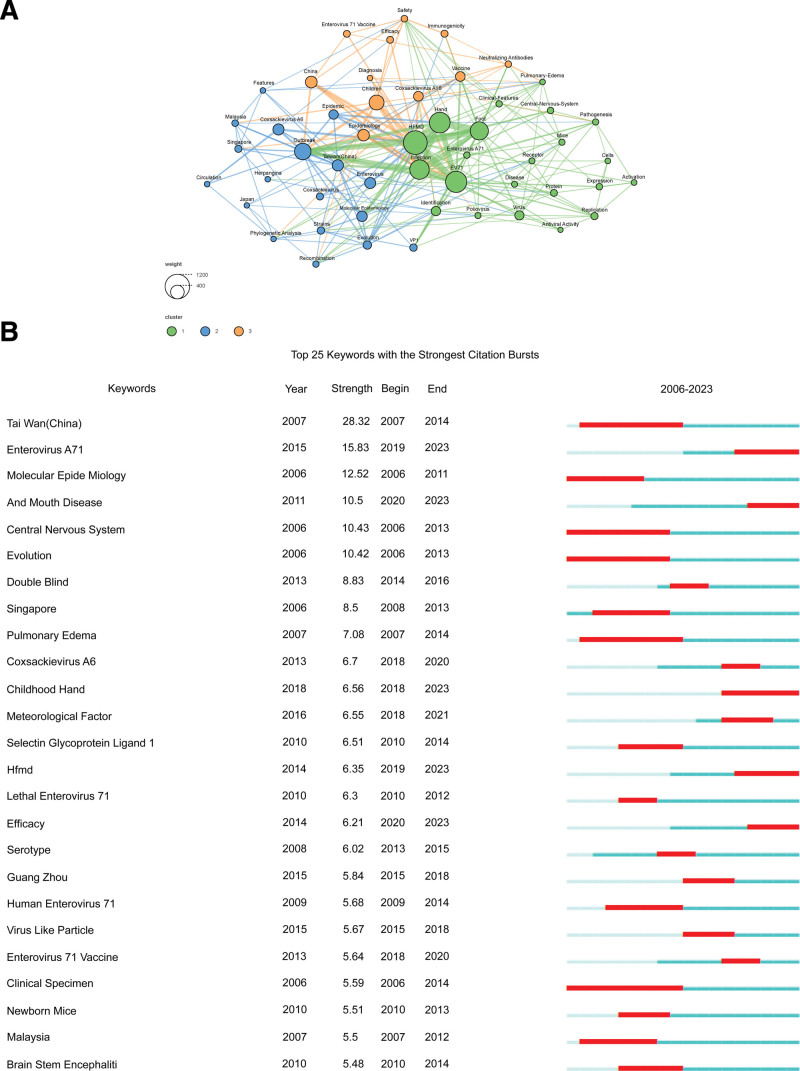
Analysis of keywords of HFMD publications. (A) HFMD-related keywords that occur at least 50 times in total; (B) top 25 keywords with the highest intensity of HFMD outbreaks.

In this study, we analyzed 4451 keywords in 1860 articles, focusing on keywords that appeared at least 50 times. As shown in Figure 6A, the co-occurrence map reveals 3 clusters: the orange cluster mainly involves vaccine research of HFMD, including 11 keywords; the blue cluster mainly focuses on epidemiological research, including 18 keywords; and the green cluster is mainly related to the pathogenesis of HFMD, including 22 keywords. Items in a cluster have a high homogeneity.^[17]^ Over the years, these fields have become the main focus of HFMD research. We also identified the top 25 keywords with the strongest burst intensity (Fig. 6B), which can help researchers identify research hotspots and determine trends. Taiwan (China) had the highest burst intensity (28.32) and duration (8 years), followed by Enterovirus A71 with a burst intensity of 15.83 and a duration of 5 years, where the thick red line represents the start and end of the outbreak. The keywords focused on the pathogens, clinical manifestations, epidemiology, and molecular biology of HFMD, which indicates that continuing to pay attention to virological, epidemiological, and clinical studies will remain hot topics in future research.

### 3.7. Article and citation analysis

**Figure 7.**
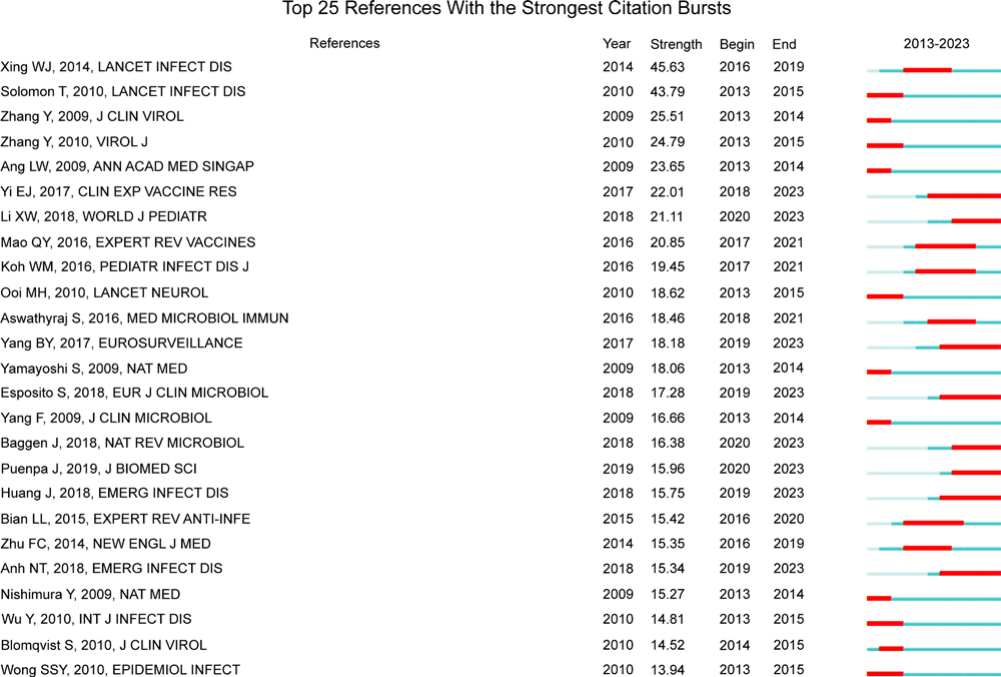
Top 25 articles with HFMD-related burst intensity.

As shown in Table 4, the most cited article is “Virology, Epidemiology, Pathogenesis, and Control of Enterovirus 71” published by Solomon Tom in 2010, and it was cited 896 times. In this study, the features of EV71 were systematically and comprehensively reviewed, which became the standard reference in the field. The article with the highest linkage strength is connected to other articles and has been frequently cited in subsequent research. Seven of the top 10 cited articles were published in Q1 journals, indicating that IF and credibility were high. Among these, the New England Journal of Medicine had the highest IF (158.5) in Q1, with an H-index of 933. The article entitled “Efficacy, safety, and immunogenicity of an enterovirus 71 vaccine in China” was mainly conducted in a randomized, double-blind, placebo-controlled, and multicenter trial. It ultimately proved the efficacy of the EV71 vaccine in preventing HFMD or herpangina associated with EV71 in infants and young children, providing important reference value for EV71 prevention and gaining widespread attention in subsequent research.^[18]^ In Figure 7, the blue line represents the time interval and the red line represents the burst duration. The first cited explosion occurred in 2013 and originated from an article published in 2010. The last 3 citation bursts occurred in 2020 and are still in progress.

**Table 4:** The top 10 high-cited references related to HFMD.

Rank	Reference	Total citations	Year	Links	Journal	IF (2023)	Quartile in category	H-index
1	Solomon T, 2010, LANCET INFECT DIS, V10, P778, DOI 10.1016/S1473-3099(10)70194-8	896	2010	350	Lancet Infect Dis	56.3	Q1	201
2	Xing WJ, 2014, LANCET INFECT DIS, V14, P308, DOI 10.1016/S1473-3099(13)70342-6	623	2014	296	Lancet Infect Dis	56.3	Q1	201
3	Ooi MH, 2010, LANCET NEUROL, V9, P1097, DOI 10.1016/S1474-4422(10)70209-X	563	2010	200	Lancet Neurol	48	Q1	259
4	Yamayoshi S, 2009, NAT MED, V15, P798, DOI 10.1038/nm.1992	372	2009	145	Nat Med	89.2	Q1	497
5	Zhang Y, 2010, VIROL J, V7, P0, DOI 10.1186/1743-422X-7-94	369	2010	188	Virol J	4.8	Q3	70
6	Zhang Y, 2009, J CLIN VIROL, V44, P262, DOI 10.1016/j.jcv.2009.02.002	314	2009	164	J Clin Virol	8.8	Q3	97
7	Ang LW, 2009, ANN ACAD MED SINGAP, V38, P106	303	2009	195	Ann Acad Med Singap	5.2	Q4	54
8	Zhu FC, 2014, NEW ENGL J MED, V370, P818, DOI 10.1056/NEJMoa1304923	300	2014	162	N Engl J Med	158.5	Q1	933
9	Wang XX, 2012, NAT STRUCT MOL BIOL, V19, P424, DOI 10.1038/nsmb.2255	300	2012	91	Nat Struct Mol Biol	16.8	Q1	249
10	Nishimura Y, 2009, NAT MED, V15, P794, DOI 10.1038/nm.1961	298	2009	125	Nat Med	82.9	Q1	497

## 4. Discussion

In this study, the literature related to HFMD published between 2006 and 2023 was analyzed by bibliometric methods. A systematic analysis was conducted on the publication trends, countries, institutions, authors, journals, keywords, and articles. Over the past 18 years, the development and spread of HFMD have seriously threatened the health and lives of children. This has led to an increasing number of researchers and institutions focusing on HFMD and conducting related research. Moreover, advances in technology have led to more accurate identification of pathogens and analysis of infectious disease outbreaks, thus promoting related research, including HFMD. The current stable research trend of HFMD is driven by the continuous epidemic of the disease and the technical progress, although this trend has been somewhat influenced by the Corona Virus Disease 2019 (COVID-19) pandemic in the past 3 years.

Since 2006, the number of articles related to HFDM has increased by more than 10 times compared to that in 2019 (Fig. 2). There are multiple reasons for this trend. First of all, with the increase in diagnosed cases of HFMD, global attention on the prevention and control of HFMD has also increased. Secondly, the development of virology and molecular biology makes the research on HFMD viruses more in-depth. In addition, public awareness of disease prevention has been continuously enhanced, and the acceleration of population movement has also led to the continuous variation of viral strains, which has promoted the research of HFMD in countries all over the world. Therefore, the number of articles related to HFMD has shown an increasing trend over the years.

Among the 64 countries participating in the HFMD research, China is the most productive and influential country, which plays a significant role in HFMD research (Fig. 3). Owing to the widespread occurrence of HFMD in China, many human and financial resources have been allocated for this research. Therefore, there are prolific authors and institutions in China, resulting in a very high output of articles. Through extensive research, China has accumulated a lot of data and experience and has a certain degree of understanding of the pathogenesis, transmission, prevention, and treatment of the disease. Meanwhile, China actively participates in international cooperation and communication, which further enhances its influence.^[19]^ International cooperation facilitates the accurate exchange of information, which can more directly promote the output of articles and research results, identify different pathogenic viruses and mutant strains in time, and exchange diagnosis and treatment suggestions in time. However, although China has the highest number of published articles, the average citations per article are lower than those of the UK, Australia, and Malaysia, indicating that some individual publications in China have a lower impact. More influential and high-quality publications are needed, and virus research in China should be further strengthened to achieve this goal. From a global perspective, the establishment of cross-national and regional cooperation is conducive to the dissemination of information and the exchange of science and technology, which is conducive to understanding and combating unknown areas related to viruses as soon as possible. With the continuous expansion of virus research around the world, more and more research institutions are involved. We can better understand the disease information, overcome difficulties, and share information on a global scale.

According to Figure 4, the most prolific institutions and authors are all from China, which proves its role and contribution as a major country. The Chinese Academy of Sciences and the Chinese Center for Disease Control and Prevention are the most prolific and influential institutions (Table 1). As the foremost academic institution in China for natural sciences, the Chinese Academy of Sciences has played a vital role in publishing a wide range of articles. The Chinese Center for Disease Control and Prevention is mainly engaged in disease prevention and control, emergency response to public health emergencies, etc, and plays an irreplaceable role in protecting citizens’ health.^[20]^ Although the number of articles published by the Chinese Center for Disease Control and Prevention is less than that of the Chinese Academy of Sciences, their influence is much stronger than that of the Chinese Academy of Sciences. Although the number of publications, H-index, and TLS of the University of Oxford were not as high as those of the Chinese Center for Disease Control and Prevention and the Chinese Academy of Sciences, the average number of citations of each article was the highest. This also indirectly reflects an institution’s influence, indicating that an institution’s influence cannot be judged only by the quantity of its publications. When investigating the influence of an institution, we must consider the quality and quantity of its articles. This indicates that there are other factors besides the number of publications in determining the impact of articles, and research institutions should focus more on article quality and research innovation. The difference in influence between China’s Disease Prevention and Control Centers and the Chinese Academy of Sciences in terms of publishing HFMD-related articles can also be attributed to their divergent focuses. The former emphasizes epidemiology and clinical diagnosis and treatment, while the latter may pay more attention to the in-depth study of disease mechanisms and the development of drugs and vaccines for treating HFMD.

In the author analysis, Zhang Yong was the most prolific author in the field of HFMD (Table 2). However, when considering a comprehensive assessment of high-impact authors, Xu Wenbo stands out from the others. (Fig. 4B). Identifying high-producing authors helps evaluate their status and influence in the academic field. High-yield authors usually cooperate with high-impact authors to promote progress in HFMD research. Highly productive authors who publish many articles quickly can represent specific research directions and hotspots, inspire other researchers to accurately and quickly identify the research focus of HFMD, and promote the development and progress of this field.

Identifying high-producing journals is an important research direction in bibliometrics. Its significance lies not only in academic evaluation and ranking, but also in providing guidance and reference for academic research, supporting policy-making and decision-making, promoting academic communication and collaboration, and promoting the development and innovation in HFMD.^[21]^ Our research findings indicate that in recent years, there have been several published articles on HFMD in specialized journals in the field of virology (Fig. 5 and Table 3), which suggests that the emphasis in HFMD-related studies has been on investigating the characteristics of disease-causing pathogens and prevention and treatment measures. High-producing journals usually have higher IFs and citation frequencies, which can be a reference for researchers when selecting journals to submit their work.

The analysis of keywords also revealed a close relationship among the topics of HFMD. In the visualization of keywords (Fig. 6A), a keyword network composed of 3 clusters is found. According to these findings, virology, molecular biology and epidemiology, clinical diagnosis and treatment, prevention, and control are the main research fields in HFMD. The emergence of keywords is helpful to understand the current and future research trends in HFMD. Future research should focus on the biological characteristics, transmission mechanisms, risk factors, epidemiology, immune research, clinical efficacy, and specific treatment methods of the virus, to better prevent and control HFMD (Fig. 6B). However, although there are some vaccines and treatments for HFMD, there is still a need to develop specific treatments and rapid diagnostic methods. Future research should focus on the rapid clinical diagnosis and specific treatment methods of HFMD, to better prevent and control it. The morbidity and mortality of HFMD remain high worldwide. The epidemiological features and trends of HFMD may continue to be the focus of research, such as age and geographical distribution, seasonality, and climate change, to formulate and implement better prevention and control strategies.^[22]^ The occurrence and spread of HFMD are closely related to the immune system, such as cellular immunity and humoral immunity. Vaccine research is still the most concerning issue in the world. Accelerating the development of effective vaccines is necessary to prevent and control HFMD.^[23]^

In 2010, Solomon and Tom published a paper on “Virology, epidemiology, pathogenesis, and control of enterovirus 71” in the famous journal “The Lancet Infectious Diseases” (Table 4). Among the 1860 articles, this article is highly cited for its comprehensive report on the hot and cutting-edge human enterovirus 71 (EV71) issues, including virology, epidemiology, pathogenesis, and control methods. This study provides essential information for future researchers, and its reliability and methods contribute to its influence. The article’s impact encourages global researchers to leverage their creativity, explore unknown information in the field, and contribute to studying HFMD. The 3 articles that began to break out in recent years focused on enteroviruses, HFMD, and the outbreaks of EV71 in the Asia-Pacific region (Fig. 7). The recent surge in interest and research on this subject can be attributed to several factors, namely, Increase in the incidence and severity of enterovirus infections. Over the past few years, there has been a surge in the number and severity of enterovirus infections, particularly in the Asia-Pacific region.^[24]^ This trend urges people to pay more attention to understanding these viruses’ life cycles and epidemiology and to formulate practical guidelines for diagnosis and treatment.^[25,26]^ The emergence of new strains: Enterovirus mutates rapidly, and new strains can appear soon. Consequently, ongoing surveillance and research are required to understand the genetic diversity of these viruses and how they may evolve. “Growing public health concerns: The increase in the incidence and severity of enterovirus infections” have led to growing public health concerns about these viruses. This urges researchers, healthcare providers, and policymakers to pay more attention to formulating effective prevention and control strategies. The recent surge in interest in and research on enterovirus can be attributed to many factors, including new strains, increased incidence and severity of infections, and growing public health concerns. The 3 articles analyzed provide insights into the enteroviruses’ life cycle, diagnosis, treatment, and epidemiology. They can provide future research and public health work on controlling these infections.

There are some limitations. First of all, the analysis only included articles from the WoSCC and may ignore articles from other databases. Additionally, excluding non-journal and review articles during article screening may overlook other viewpoints. The article focuses on HFMD but pays little attention to viruses that cause the disease, which leads to an incomplete conclusion.

## 5. Conclusion

This study analyzed article metrics to examine publications related to HFMD and identified the most prolific journals, authors, and countries in this field. In future research, it would be beneficial to determine the current and potential future research hotspots, which can provide researchers with a more comprehensive range of choices. Our findings indicate that China is the most productive country. The Chinese Academy of Sciences is the most prolific institution, while the Chinese Center for Disease Control and Prevention is the most influential institution. China has a high prevalence rate of HFMD, making it a leading region for HFMD research. Our research will provide valuable information related to HFMD, and help researchers to detect and prevent this disease at an early stage.

## Author contributions

**Conceptualization:** Ni Zhu.

**Data curation:** Yunzhi Li.

**Formal analysis:** Yunzhi Li.

**Funding acquisition:** Yujie Xiao, Jiawei Liang, Ni Zhu.

**Investigation:** Junjie Ye.

**Methodology:** Yunzhi Li, Xiangjie Zhai, Junjie Ye, Yujie Xiao, Jiawei Liang.

**Project administration:** Yunzhi Li.

**Validation:** Yujie Xiao, Jiawei Liang.

**Visualization:** Xiangjie Zhai, Junjie Ye.

**Writing – original draft:** Yunzhi Li, Ying Ruan.

**Writing – review & editing:** Ni Zhu.

## References

[1] Xing J Wang K Wang G Li N Zhang Y. Recent advances in enterovirus A71 pathogenesis: a focus on fatal human enterovirus A71 infection. Arch Virol. 2022;167:2483–501. 36171507 10.1007/s00705-022-05606-4

[2] Zhu P Ji W Li D. Current status of hand-foot-and-mouth disease. J Biomed Sci. 2023;30:15. 36829162 10.1186/s12929-023-00908-4 PMC9951172

[3] Ismail A Saahath A Ismail Y Ismail MF Zubair Z Subbaram K. “Tomato flu” a new epidemic in India: virology, epidemiology, and clinical features. New Microbes New Infect. 2022;51:101070. 36582550 10.1016/j.nmni.2022.101070 PMC9792351

[4] Bello AM Roshorm YM. Recent progress and advances towards developing enterovirus 71 vaccines for effective protection against human hand, foot and mouth disease (HFMD). Biologicals. 2022;79:1–9. 36089444 10.1016/j.biologicals.2022.08.007

[5] Yang J Wu J Han T. Global research hotspots and frontiers of myasthenia gravis from 2002 to 2021: a bibliometric study. Medicine (Baltim). 2023;102:e34002. 10.1097/MD.0000000000034002 PMC10270528 37327308

[6] Hou Z Wang W Su S. Bibliometric and visualization analysis of biomechanical research on lumbar intervertebral disc. J Pain Res. 2023;16:3441–62. 37869478 10.2147/JPR.S428991 PMC10590139

[7] Chen CM. CiteSpace: A practical Guide for Mapping Scientific Literature. New York: Nova Science Publishers Hauppauge. 2016.

[8] Chen CM. Citespace II: detecting and visualizing emerging trends and transient patterns in scientific literature. J Am Soc Inf Sci Technol. 2005;57:359–77.

[9] Zhang J Song L Xu L. Knowledge domain and emerging trends in ferroptosis research: a bibliometric and knowledge-map analysis. Front Oncol. 2021;11:686726. 34150654 10.3389/fonc.2021.686726 PMC8209495

[10] Wu X Deng Z Zhao Q. Immunotherapy improves disease prognosis by affecting the tumor microenvironment: a bibliometric study. Front Immunol. 2022;13:967076. 36275770 10.3389/fimmu.2022.967076 PMC9582136

[11] Ren D Lee B Brehmer M. Charticulator: interactive construction of bespoke chart layouts. IEEE Trans Vis Comput Graph. 2019;25:789–99. 10.1109/TVCG.2018.2865158 30136992

[12] Yang T Zhang Z Zhu W Meng LY. Quantitative analysis of the current status and research trends of biochar research – a scientific bibliometric analysis based on global research achievements from 2003 to2023. Environ Sci Pollut Res Int. 2023;30:83071–92. 37338685 10.1007/s11356-023-27992-1

[13] Aria M Cuccurullo C. Bibliometrix: an r-tool for comprehensive science mapping analysis. J Informetrics. 2017;11:959–75.

[14] Li F Zhang D Chen J Tang K Li X Hou Z. Research hotspots and trends of brain-computer interface technology in stroke: a bibliometric study and visualization analysis. Front Neurosci. 2023;17:1243151. 37732305 10.3389/fnins.2023.1243151 PMC10507647

[15] Lu H Han T Li F Yang J Hou Z. Global trends and hotspots in research of robotic surgery in oncology: a bibliometric and visual analysis from 2002 to 2021. Front Oncol. 2022;12:1055118. 36439475 10.3389/fonc.2022.1055118 PMC9691977

[16] Hou Z Jiang P Su S Zhou H. Hotspots and trends in multiple myeloma bone diseases: a bibliometric visualization analysis. Front Pharmacol. 2022;13:1003228. 36313356 10.3389/fphar.2022.1003228 PMC9614215

[17] Castejón-de la Encina ME Delgado Sánchez R Ayuso Baptista F López Mesa F Castro Delgado R. Presentation of the prehospital emergency research network and an analysis of bibliometric indicators for scientific productivity in out-of-hospital care. Emergencias. 2022;34:213–9. 35736526

[18] Li C Geng M Li S. Knowledge mapping of surgical smoke from 2003 to 2022: a bibliometric analysis. Surg Endosc. 2024;38:1465–83. 38228836 10.1007/s00464-023-10641-6 PMC10881617

[19] Fei X Wang S Zheng X Liu K Liang X. Global research on cognitive behavioural therapy for schizophrenia from 2000 to 2019: a bibliometric analysis via CiteSpace. Gen Psychiatr. 2021;34:e100327. 33585791 10.1136/gpsych-2020-100327 PMC7845669

[20] Zhang X Zhang Y Li H Liu L. Hand-foot-and-mouth disease-associated enterovirus and the development of multivalent HFMD vaccines. Int J Mol Sci. 2022;24:169. 36613612 10.3390/ijms24010169 PMC9820767

[21] Lan D Huang C Yu N Lao J Li Z. Research trends of acupuncture therapy on facial paralysis in a decade spanning 2013–2023: a bibliometric analysis. Complement Ther Med. 2023;79:103006. 37972694 10.1016/j.ctim.2023.103006

[22] Nayak G Bhuyan SK Bhuyan R Sahu A Kar D Kuanar A. Global emergence of Enterovirus 71: a systematic review. Beni Suef Univ J Basic Appl Sci. 2022;11:78. 35730010 10.1186/s43088-022-00258-4 PMC9188855

[23] Zheng D Shen L Wen W. The impact of EV71 vaccination program on hand, foot and mouth disease in Zhejiang Province, China: a negative control study. Infect Dis Model. 2023;8:1088–96. 37745754 10.1016/j.idm.2023.09.001 PMC10514095

[24] Puenpa J Wanlapakorn N Vongpunsawad S Poovorawan Y. The history of enterovirus A71 outbreaks and molecular epidemiology in the Asia-Pacific region. J Biomed Sci. 2019;26:75. 31627753 10.1186/s12929-019-0573-2 PMC6798416

[25] Baggen J Thibaut HJ Strating JRPM van Kuppeveld FJM. The life cycle of non-polio enteroviruses and how to target it. Nat Rev Microbiol. 2018;16:368–81. 29626210 10.1038/s41579-018-0005-4

[26] Li XW Ni X Qian SY. Chinese guidelines for the diagnosis and treatment of hand, foot and mouth disease (2018 edition). World J Pediatr. 2018;14:437–47. 30280313 10.1007/s12519-018-0189-8

